# Polymorphisms involving gain or loss of CpG sites are significantly enriched in trait-associated SNPs

**DOI:** 10.18632/oncotarget.5650

**Published:** 2015-10-14

**Authors:** Dan Zhou, Zhenli Li, Dan Yu, Ledong Wan, Yimin Zhu, Maode Lai, Dandan Zhang

**Affiliations:** ^1^ Department of Pathology, Zhejiang University School of Medicine, Hangzhou, Zhejiang, 310058, China; ^2^ Key Laboratory of Disease Proteomics of Zhejiang Province, Hangzhou, Zhejiang, 310058, China; ^3^ Department of Epidemiology & Biostatistics, Zhejiang University School of Public Health, Hangzhou, Zhejiang, 310058, China

**Keywords:** epigenetic, DNA methylation, cancer, single nucleotide polymorphism (SNP), CpG site

## Abstract

Some single nucleotide polymorphisms (SNPs) influence the existence of CpG sites, the basis of DNA modification such as methylation and hydroxymethylation. These polymorphisms can lead to gain or loss of CpG sites and were defined as CpG site related SNPs (cgSNPs) in this study. The cgSNPs change DNA sequence and might potentially affect DNA modification such as methylation. However, the functional consequence of cgSNPs is poorly understood. We observed that a considerable proportion (23.0%) of common variants were cgSNPs in human genome. Mutations involving loss of CpG sites were associated with reduced levels of methylation (~20.2%) using The Cancer Genome Atlas (TCGA) data. Using public databases (SCAN and seeQTL) of expression quantitative trait loci (eQTLs), we found that the cgSNPs were significantly enriched in eQTLs via logistic regression and simulation test. Furthermore, we observed that cgSNPs were more likely to be trait-associated loci especially cancers using a catalog of published genome-wide association studies (GWAS) recorded by National Human Genome Research Institute (NHGRI). Our results indicated that cgSNP might be meaningful as annotation either in SNP functional prediction or in screening for trait-associated SNPs.

## INTRODUCTION

Individual genetic variants contribute to phenotypic variants and disease susceptibility. Single nucleotide polymorphisms (SNPs) are the most common type of genetic variation in human genome. Of those, some SNPs that influence CpG dinucleotides, which can generate or abolish a CpG site. For example, a C-to-T transition on ‘C’ of CpG dinucleotides leads to a loss of a CpG site. Here, we defined these variants as CpG site related SNPs (cgSNPs).

In mammals, the majority of cytosines (70%–80%) in CpG dinucleotides are methylated in somatic cells. [1, 2] DNA methylation has been evidently linked to transcriptional regulations [3]. Differentially methylated regions (DMRs) have been focused by numerous studies in complex diseases. The role of DNA methylation in cancer etiology and progression is well established [4, 5].

Besides the sequence changes, cgSNPs might potentially affect DNA modifications. Several studies have explored DNA methylation associated SNPs in various human tissues and cell lines [6–8]. Hundreds of methylation quantitative trait loci (mQTLs) have been reported using high-throughput data [9–11]. The mQTL studies have predominantly focused on the mapping of methylation of CpG sites without harboring DNA sequence variants. However CpG sites containing SNPs were rarely explored because the methylation levels of CpG sites involving SNPs were not covered by most methylation detection platforms [11–14]. Until recently, Degui Zhi and his colleagues have focus on cgSNP and they observed that cgSNPs account for over two thirds of the strongest mQTL signals [15]. However, the biological relevance of cgSNP was still poorly understood.

We raised the question from the fundamental changes of DNA sequence and its putative effect in epigenetics. Given the essential role of DNA modification in the regulation of gene expression, what are the functional consequence of cgSNPs in gene expression and complex diseases? More specifically, the question we might ask: Are cgSNPs more likely to be enriched in eQTLs and trait-associated variants? We therefore performed this study to test the hypothesis that cgSNPs enriched in eQTLs and trait-associated variants. Firstly, we identified cgSNPs in human genome from HapMap phase II dataset. Then we retrieved eQTLs and trait-associated SNPs from online databases. Finally, we utilized logistic regression and permutation test to assess whether cgSNPs were enriched in eQTLs and trait-associated SNPs.

## RESULTS

### General descriptions of cgSNPs in genome

We obtained 4,097,556 SNPs from HapMap phase II dataset. Of those, 942,429 loci (23.0%) were cgSNPs. There were 42 scenarios of single base substitution that could cause gain or loss of a CpG site (Supplementary Table 1). 80.7% of cgSNPs were attributable to A/G or C/T substitutions in eight trinucleotides including CRT, CRG, CRC, CRA, AYG, CYG, GYG and TYG (R and Y were the International Union of Pure and Applied Chemistry (IUPAC) code which refers to A or G and C or T respectively). The proportions of cgSNPs among all the variants varied in different chromosomes ranging from 20.9% to 27.9%. 365098 and 9008 SNPs were found to be cgSNPs located in genebodies and promoters respectively.

According to the ancestral sequence, cgSNPs were classified into cg-gain-SNPs and cg-loss-SNPs. There were 489,891 (52.0%) cg-gain-SNPs and 449,796 (47.9%) cg-loss-SNPs. Due to the lack of ancestral sequence, a very small proportion of cgSNPs could not be classified as cg-gain-SNPs or cg-loss-SNPs. We observed that cg-loss-SNPs accounted for a major proportion (69.0%) of cgSNPs located in CpG islands (Table 1). The proportion of cg-loss-SNPs in CpG island shores was 48.7%, which was similar to the open seas (47.6%).

**Table 1:** The distribution of cg-gain-SNP and cg-loss-SNP located in CpG island, CpG island shore and open sea

Locations	cgSNP*	cg-gain-SNP (%) ^#^	cg-loss-SNP (%) ^#^
CpG island	4486	1300 (29.0)	3095 (69.0)
CpG island shore	40104	20391 (50.8)	19542 (48.7)
Open sea	897839	468200 (52.1)	427159 (47.6)
Global	942429	489891 (52.0)	449796 (47.7)

* The number of cgSNP in each region including cg-gain-SNP, cg-loss-SNP and a fraction of cgSNPs which could not be classified due to the lack of ancestral sequence.

# The number of cg-gain-SNPs or cg-loss-SNPs and the proportions (%) of cg-gain-SNP and cg-loss-SNP in cgSNPs located in each region.

### Loss of CpG site was associated with reduced level of methylation

**Figure 1:**
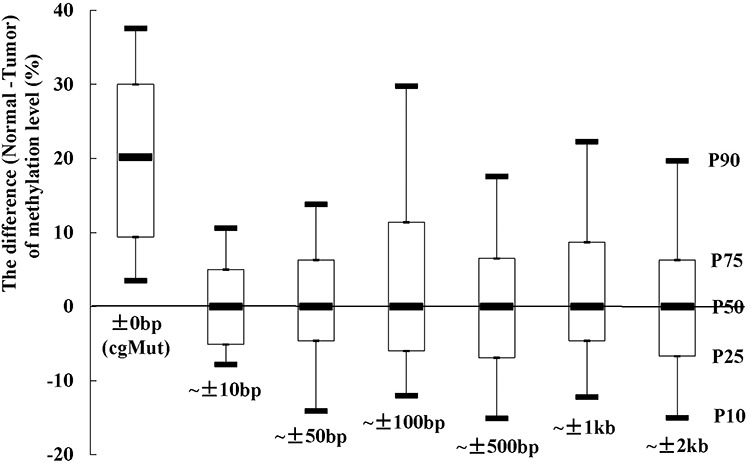
The methylation levels at CpG sites, which existed in normal tissue sample and lost in paired tumor sample due to mutations (cgMut), were higher in normal than tumor tissue. While no differences of methylation levels of nearby CpG sites were observed The differences of methylation levels between normal and paired tumor tissue sample (TCGA ID = 3518) in cgMut related and nearby CpG sites (classified into 6 bins including ~ ± 10bp, ~ ± 50bp, ~ ± 100bp, ~ ± 500bp, ~ ± 1kb and ~ ± 2kb away from cgMut) were presented as box plot of the 50^th^ percentile (P50, Median) and range of difference of methylation levels. The top and bottom of the box represent the 75^th^ and 25^th^ percentile. The whiskers indicate the 10^th^ and 90^th^ percentile. An example was given in ~ ± 2kb bin.

Totally 53 somatic exon mutations, observed in tumor tissue of the colon cancer sample in TCGA, could lead to loss of CpG sites (Supplementary Table 2). The methylation levels were lower in tumor tissue in 94.3% (50 of 53) of the CpG sites compared with paired normal tissue. The 25^th^ percentile, Median and 75^th^ percentile of the difference of methylation levels (normal tissue minus tumor tissue) were 9.4%, 20.2% and 30.0%, respectively. We compared the methylation levels of the nearby CpG sites as well. As shown in Figure 1, the methylation levels of nearby CpG sites (within 10bp away from the cgMuts (mutations involving gain or loss of CpG sites)) showed no difference between tumor and paired normal tissue sample (the Median of difference was 0%). We also observed that all of the Medians of the differences in the other 5 bins (including ~ ± 50bp, ~ ± 100bp, ~ ± 500bp, ~ ± 1k and ~ ± 2k bins) were 0%.

### cgSNPs were significantly enriched in eQTLs

**Figure 2:**
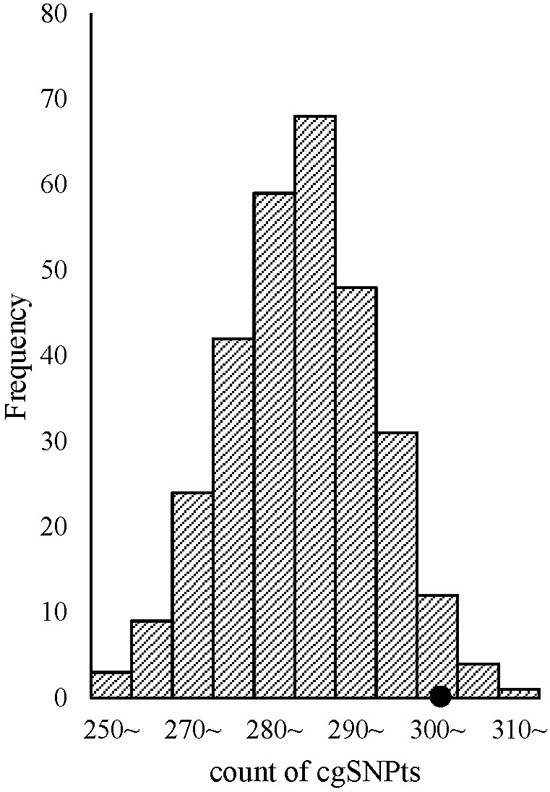
cgSNPts are significantly enriched in eQTLs The theoretical distribution of the counts of cgSNPts in 300 draws (each draw containing 500 SNPs, which is matched to the 500 eQTLs) is shown in histogram. The observed count of cgSNPts in the 500 eQTLs was 303 (shown as a solid circle). According to the observed count of cgSNPts and the distribution of the counts of cgSNPts in 300 matched draws, the enrichment *P*-value was 0.04.

Significant enrichment of cgSNPs in eQTLs was observed from logistic regression after adjusting for covariates including MAF and the number of SNPs which could be tagged by the tested proxy SNP (see methods). The OR and its 95%CI of cgSNPt (a cgSNP or a non-cgSNP but could tag at least one cgSNP) was 1.58 (1.48~1.69). In simulation tests, 303 out of 500 eQTLs with top signals were cgSNPts (Figure 2), which was significantly higher than matched SNP sets from 300 times stratified random sampling (*P* = 0.04).

Then we conducted logistic regressions with SNPs which located near (including islands and shores) or distant from CpG islands separately. Results showed that the ORs of cgSNPts near CpG islands were 1.67 (95%CI: 1.39~2.00) and 1.57 (95%CI: 1.47~1.68) for cgSNPs distant from CpG islands. Consistent results were obtained from simulation tests.

### cgSNPs were significantly enriched in trait-associated SNPs especially cancers

**Figure 3:**
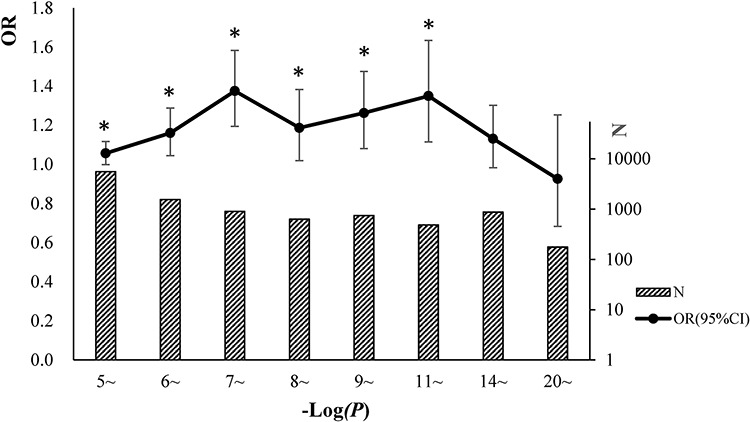
Trait-associated loci (all the traits were pooled) were significantly associated with cgSNPts We classified the trait-associated loci into 8 bins according to the reported *P*-values and did analyses for each bin. The numbers of trait-associated loci in 8 bins were shown in slash bar graphs. The solid circles indicated the ORs of cgSNPts from logistic regressions. The whiskers represented 95% confidence intervals (CI) of ORs. A ‘*’ was marked if the logistic regression of enrichment test achieved statistical significance in each bin (*P* < 0.05).

**Figure 4:**
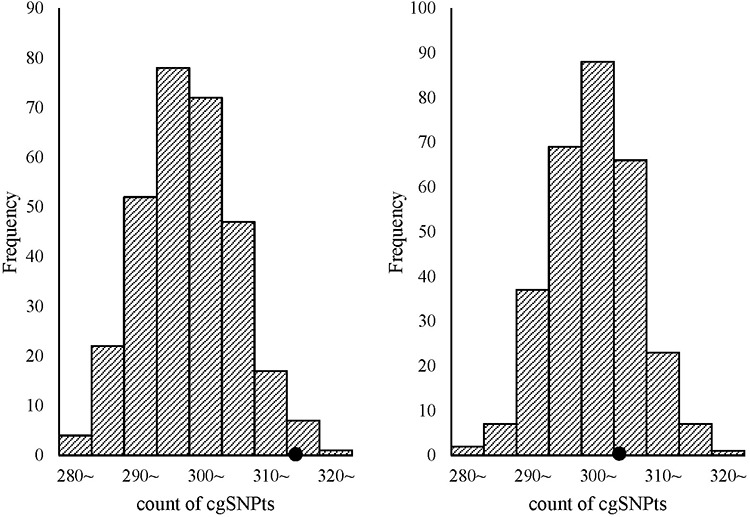
cgSNPts are significantly enriched in trait-associated loci with the reported *P*-values ranging from E-5~ to E-11~, while no enrichment was observed with *P*-values less than E-14 The theoretical distributions of the count of cgSNPts in 300 draws (each draw containing 500 SNPs, which is matched to the 500 trait-associated loci) are shown in the histograms. The observed counts of cgSNPts in the 500 trait associated-loci are shown as solid circles. The left graph shown that cgSNPts were significantly enriched in trait-associated loci with *P*-values ranging from E-5~ to E-11~ (count = 317/500, *P* = 0.023), while the right graph indicated that cgSNPts were not enriched in trait-associated SNPs with *P*-values less than E-14 (count = 306/500, *P* = 0.31).

Trait-associated SNPs (from the catalog of reported loci via GWA studies) with reported *P*-values less than E-5 were classified into 8 bins according to the reported *P*-values (Figure 3). The associations between cgSNPt and the trait-associated SNP were conducted in each bin via logistic regressions adjusted for covariates including MAF and the number of SNPs tagged by the tested proxy SNP (see more details in methods). The results showed that trait-associated SNPs with reported *P*-values ranging from E-5~ to E-11~ were more likely to be cgSNPts compared with other SNPs. However, these associations were not observed in the rest two bins with the top signals. The number (N) of trait-associated SNPs, the OR and its 95%CI of cgSNPts in each bin were presented in Figure 3. Simulation tests got consistent results with logistic regressions (Figure 4), showing that the cgSNPts were significantly enriched in trait-associated loci with reported *P*-values ranging from E-5~ to E-11~. Enrichment was not observed in trait-associated loci with reported *P*-values less than E-14.

SNP function predictions were performed for each trait-associated SNP to explore the unique features of those trait-associated SNPs without enrichment signals. Results suggested that each SNP from the top two bins (with the strongest trait associations) could averagely tags (LD r^2^ > 0.8) 1.39, 0.30, 0.28 and 0.21 SNPs which predicted to be transcription factor binding sites, splicing related variants, miRNA binding sites and non-synonymous mutations, respectively. While the mean numbers of SNPs with potential functions tagged by trait-associated SNPs from the rest six bins with *P*-values E-5~ to E-11~ were 0.79, 0.13, 0.07 and 0.06, respectively.

We did analyses in cgSNPs near CpG islands and cgSNPs located in open sea separately. Results showed that the ORs for cgSNPts near CpG islands was 1.16 (95%CI: 1.05~1.29) and 1.12 (95%CI: 1.08~1.15) for cgSNPs in open sea. Consistent results obtained from simulations as well.

**Figure 5:**
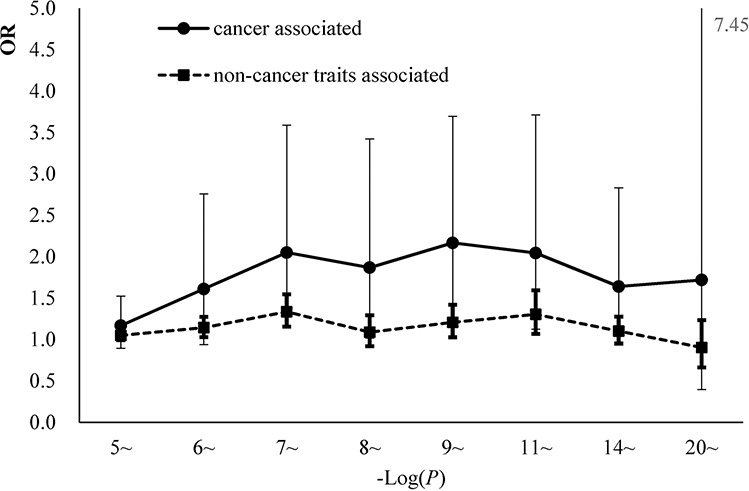
The comparisons of the enrichment effect sizes between cgSNPts in cancer associated loci and non-cancer associated loci The OR and 95% CI were revealed by logistic regressions in 8 bins (according to the reported *P*-values). The effect sizes of the associations between cancer associated loci and cgSNPts were marked with solid circles. The effect sizes of the associations between non-cancer trait-associated loci and cgSNPts were marked with solid squares.

**Figure 6:**
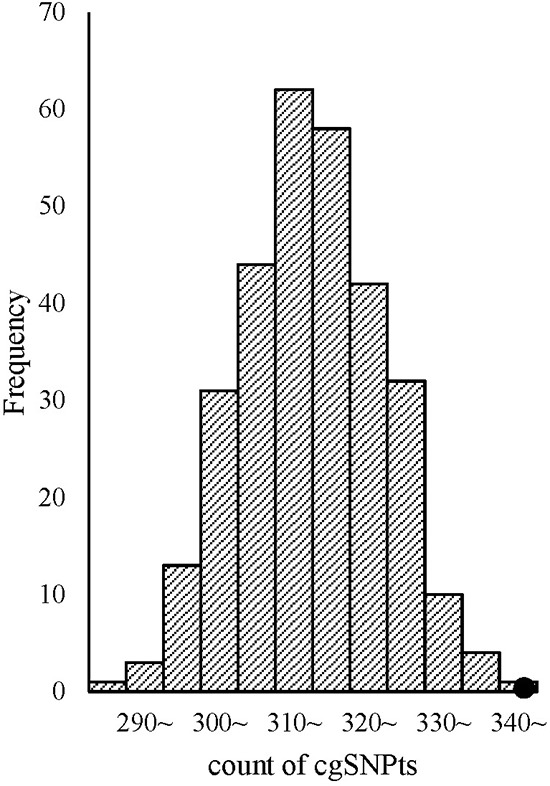
cgSNPts are significantly enriched in cancer associated loci The theoretical distributions of the number of cgSNPts in 300 draws (each draw containing 500 SNPs, which is matched to the 500 cancer associated loci) are shown in the histogram. The observed count of cgSNPts in the 500 cancer associated loci is 345 (shown as a solid circle). According to the observed count of cgSNPts and the distribution of the counts of cgSNPts in 300 matched draws, the enrichment *P*-value was less than 0.003.

Additionally, trait-associated SNPs were classified into three categories, according to the ‘Disease/Trait’ label in the catalog of GWAS in NHGRI, including obesity-associated variants, cancer-associated variants, neurological disease associated variants etc. We observed that the effect sizes of obesity and neurological associated sub-categories were similar to pooled category. However in cancer associated category, the logistic regression revealed significant larger effect than other categories. OR for cgSNPt was 1.50 (95%CI 1.26–1.77) for cancer associated category compared with 1.11 (95%CI 1.06–1.15) in other trait-associated category (Figure 5). We performed the simulation test in 500 cancer associated SNPs (Figure 6). Similarly, the effect of cgSNPt was more pronounced in cancer associated loci (*P* < 0.003).

### Supplementary analyses

We used varied threshold of LD-pruning to assess the enrichment of cgSNPt in trait-associated loci. The effect of cgSNPt remained stable when the threshold of LD-pruning varied from r^2^ =0.8 (O*R* = 1.12 *P* = 1.1E-8) to r^2^ = 0.5 (O*R* = 1.12 *P* = 1.6E-6) and r^2^ = 0.3 (O*R* = 1.11 *P* = 6.4E-4). Enrichment analyses were performed only using CEU-based LD data and association studies from samples of European ancestry. Consistent results of the enrichment analysis were obtained (Supplementary Figure 1).

## DISCUSSION

In this study, we identified cgSNPs in human genome and assessed their biological relevance. We observed that approximately a quarter of SNPs are cgSNPs in human genome. A higher proportion of cgSNPs involving loss of CpG sites was found in CpG islands. Logistic regressions and simulation tests revealed that cgSNPs were enriched in eQTLs and trait-associated SNPs especially in cancers. Enrichments were observed for cgSNPs located near CpG islands, as well as cgSNPs located distant from CpG islands.

It is well accepted that the existence of CpG sites is an essential prerequisite of DNA modifications. While, to our knowledge, few studies paid attention to SNPs involving CpG sites whether they would be a potential biomarker which influence the epigenetic modification directly [15]. Recently, a trait-associated cgSNP was reported by a genome-wide association study on the metabolism of methionine [16]. This study showed a genotype-methylation-phenotype three-way association. cgSNP rs11752813 simultaneously showed significant association with both DNA methylation and the difference between pre- and post-methionine load test tHcy levels (ΔPOST). The methylation level on this CpG site was significantly associated with phenotypes (ΔPOST) after controlling for the genotype of the cgSNP. This result supported the hypothesis that cgSNPs could be functional via creating or eliminating a CpG site. Allele-specific methylation patterns, which associated with the overall- and disease-specific survival of diffuse large B-cell lymphoma, were observed in a recent study [17].

The present study assessed the role of cgSNPs in a genome-wide level. Of cgSNPs, cg-gain-SNPs and cg-loss-SNPs are almost equally distributed. It is reported that the mutation rate is significantly increased in low-intermediately (20–40% methylation level) to intermediately methylated CpG sites (40–60% methylation level) of human genome [18]. CpG sites in CpG islands (CGI) typically show hypomethylation, whereas CpG sites in non-CGI regions exhibit hypermethylation as summarized by Jones et al [19]. The difference of methylation levels may partly account for the higher proportion of cg-loss-SNPs in CGI.

The decreased methylation levels of the cg-loss-mutations were observed using data from TCGA. The methylation levels of the 53 CpG sites were considerably higher in normal tissue than paired tumor tissue sample that lost CpG sites because of mutations. It should be noted that the observed difference levels of methylation (20.2% averagely) may be attributable to the mutations as well as the difference between tumor and normal tissue. However, the methylation levels were similar between cancer and normal tissues in CpG sites (~ ± 10bp to ~ ± 2kb bins) approximate to the cgMuts (Figure 1). That means the methylation difference we found were probably resulted from these point mutations rather than the tissue difference. As CpG sites harboring cg-gain-SNPs were not considered as CpG sites according to the refSeq, the methylation levels on CpG sites involving cg-gain-SNPs were not available for analyses. Although the methylation measurements at cg-gain-SNPs were not directly available, we speculated that they probably have similar effects as those observed cg-loss-SNPs since cg-gain-SNP and cg-loss-SNP were defined according to ancestral alleles.

The enrichment of cgSNPts in eQTLs indicated that cgSNPs contributed to the regulation of gene expression. Besides DNA sequence change, cgSNPs are potentially associated with DNA modifications. It is well acknowledged that DNA modification especially methylation plays a vital role in gene expression. The enrichment of cgSNPts in eQTLs supported the tight correlation between DNA modification and gene expression.

We further observed that cgSNPs either located near CGI or distant from CGI were both enriched in eQTLs. The effect size was a little bit larger in cgSNPs located near CGIs, while the difference was limited (1.67 v.s. 1.57). Our results indicated that, globally, cgSNPs had an impact on gene expression. These findings suggested that enrichment of cgSNPs in eQTLs was not limited to cgSNPs in CpG islands or island shores which had been demonstrated as faithful locations with methylation related biomarkers [20, 21].

After controlling for potential confounders, the results of logistic regressions and simulation tests suggested that cgSNPts were enriched in phenotype-associated variants. The logistic regressions showed that cgSNPts were significantly enriched in trait-associated SNPs with the reported *P*-values ranging from E-5 to E-14. However this association weakened in SNPs with reported *P*-values ranging from E-14 to E-20 and disappeared in SNPs with reported *P*-values lower than E-20. According to the data of SNP function predictions, we observed that SNPs with top signals in GWA studies had a higher probability to tag SNPs with potential functions which we already know (e.g. non-synonymous mutations). This finding indicated that the effects of these SNPs with the strongest signals in GWA studies might be independent of our hypothesis. Enrichments of cgSNPts in trait-associated loci were observed near or distal from CpG islands and distal. Results prompted that a fair amount of buried treasures located in deep open seas waited to be explored.

Given the well-established role of DNA modification in cancer, we further tested our hypothesis in cancer-associated loci compared with other traits. cgSNPt confer an increased risk of 1.5 fold for cancer-associated variants and 1.1 fold for other trait-associated SNPs. Our findings suggested that cgSNPs play an important role in cancers. Combined with the hypothesis, this finding was consistent with the general consensus that DNA modification was a critical regulator in carcinogenesis [22, 23]. Previous studies mainly focused on differently methylated patterns between tumor and normal tissues with the same DNA sequence. While another possible mechanism, indicated from our results, is that cgSNP influence the genetic susceptibility of cancers via influencing DNA modification which resulted from the property of gain or loss of CpG site.

To investigate the effect of LD in the enrichment tests, [24] we conducted additional tests in which we retain only one of any SNP set with r^2^ > 0.5 and r^2^ > 0.3 instead of r^2^ > 0.8. The results revealed that the effect sizes of cgSNPs remained stable. Considering the pooled populations with different levels of LD between SNPs may cause false positive outcomes, [25] further analyses were conducted in CEU samples. Enrichments of cgSNP in trait-associated loci were observed using data only from European ancestry populations.

The tested hypothesis in current study could be regarded as a novel annotation strategy which provided a supplementary way beyond classical annotations [26–28]. It could be considered either as a valuable clue in moving from replicated tag mutations to causal variants [29, 30] or as a priori in discovery step of association studies beyond the *P*-value [31, 32].

In conclusion, the present study highlights the biological relevance of cgSNPs. We provided novel perspective on these variants which lead to gain or loss of CpG sites directly in human genome, and evidenced that cgSNPs were significantly enriched in trait-associated SNPs especially cancers. Our findings provided a new way for SNP annotation and interpretation of association studies.

## MATERIALS AND METHODS

### Data collections

4,097,556 SNPs reported in HapMap phase II dataset were used for genome-wide cgSNP identifications. The flanking sequence was retrieved from dbSNP (see URLs) using a perl program. Single base substitution that can cause gain or loss of a CpG site is defined as cgSNP. As the methylation levels at CpG sites in close proximity are found to be highly correlated, [33] variants involving a shift of the CpG dinucleotides were not considered as cgSNPs in the present study. For example, a C-to-G transition of the second cytosine in CCG trinucleotides (i.e. 5′… C [C/G] G…3′) can lead to a gain as well as a loss of a CpG site, namely, single base shift of the CpG dinucleotides. Then, taken the ancestral trinucleotides as initial state, cgSNPs were classified into ‘cg-gain-SNPs’ if the mutations could create CpG sites and ‘cg-loss-SNPs’ if the CpG sites would be abolished.

We downloaded data of methylation levels on CpG sites harboring somatic mutations of tumor and paired normal tissue samples from TCGA (DNA methylation was detected by whole-genome bisulfite sequencing, sample ID = 3518). Mutations involving gain or loss of CpG sites (cgMut) were identified. Then we compared the differences of methylation levels of a number of CpG sites, which existed in normal tissue and lost in tumor tissue sample due to mutations (cgMut). The methylation levels of CpG sites near cgMuts were compared between tumor and paired normal tissue sample as well.

Minor allele frequency (MAF) and the number of SNPs which could be tagged by each proxy SNP were calculated via PLINK based on dataset from HapMap II with pooled populations [34]. Similar analysis was performed only using data from populations of European ancestry.

Gene coordinates and refSeq annotations were obtained from UCSC (Jul 2013 release, hg19). The position of ‘promoter’ was defined as up 2kb of the 5′ flanking regions of gene body. Coordinates of CpG islands were required from UCSC annotations (Jun 2014 release, hg19). Up and down 2kb of the CpG islands were defined as CpG island shores. Open sea regions were defined as more than 2kb distance from CpG islands in the genome [11]. Data of trait-associated SNPs were obtained from a catalog of published genome-wide association studies recorded by NHGRI (see URLs). All of the trait-associated loci were pooled together for enrichment analyses. In addition, traits were separated into different categories according to the ‘Disease/Trait’ label in the catalog of GWAS in NHGRI, including obesity-associated, cancer-associated, neurological diseases (see supplementary file for details). We also conducted the same analysis for each category.

Expression quantitative trait loci (eQTLs) were downloaded from seeQTL and SCAN database. [35, 36] The seeQTL database integrated human eQTL datasets including lymphoblastoid cell lines, human cortical samples and monocytes. SCAN collected a series of published eQTL data assayed on HapMap lymphoblastoid cell lines from 87 HapMap European descent from Utah (CEU) and 89 Yoruban from Ibadan Nigeria (YRI) samples. The overlapped *cis-*regulatory (*cis* was defined as within 1Mb of the gene) SNPs from seeQTL and SCAN were used for subsequent analysis of enrichment test of eQTLs. Linkage disequilibrium (LD) among the SNPs may affect enrichment tests, so we defined that SNPs with r^2^ > 0.8 could share the bioinformation of trait associations and eQTLs in statistical tests.

SNP function predictions (including transcription factor binding sites, splicing related variants, miRNA binding sites and non-synonymous mutations) were conducted using an online tool supported by National Institute of Environmental Health Sciences (see URLs).

As the MAF distribution for SNPs was different between all the SNPs reported in HapMap II and the SNPs used for GWAS, [24] we used 585,142 proxy SNPs from Illumina Human-OmniExpress 760k chip after LD pruning (threshold r^2^ = 0.8) for subsequent analysis. Additionally, r^2^ = 0.5 and r^2^ = 0.3 were also considered as the thresholds for LD pruning.

### Logistic regressions

We utilized a logistic regression framework to evaluate whether cgSNPs were more likely to be eQTLs or trait-associated loci. The regression model (shown below) built a linear relationship between the possibility of the proxy SNPs to be eQTLs or trait-associated loci and a group of independent variables through logit transformation.

$$\text{Model}:\ln(\text{P}/(1\text{-P}))=\beta_{0}+\beta_{\text{cgSNPt }}{\text{X}}_{\text{cgSNPt}}+\beta_{\text{MAF }}{\text{X}}_{\text{MAF}}+\beta_{\text{tags }}{\text{X}}_{\text{tags}}$$

Here, the independent variable X_cgSNPt_ represented whether a SNP belongs to cgSNPt (a cgSNP or a non-cgSNP but could tag at least one cgSNP). The binary vector X_cgSNPt_ was assigned ‘1′ if this proxy variant is a cgSNPt, assigned ‘0′ if not. The Odds ratio (OR) for cgSNPt and its 95% confidence interval (95%CI) were calculated. MAF (X_MAF_) and the count of SNPs which could be tagged by the proxy SNP (X_tags_) were considered as potential confounders and controlled as covariates in logistic regression model using SAS for Windows (version 9.2, SAS Institute Inc., Cary, NC, U.S.)

### Simulation tests

We conducted simulation tests to assess the enrichment of cgSNPs in eQTLs. Firstly, we classified all of the 585,142 proxy SNPs into different bins according to the MAF and the count of SNPs which could be tagged by the proxy SNP as matching factors in simulation test. Secondly, a list of 500 proxy SNPs, with top eQTL signals, were selected. Then, 500 SNPs were generated for 300 times via stratified random sampling (without replacement) matching stratified factors according to the 500 proxy eQTLs. Then, a distribution of cgSNPts in the 300 sets was generated as the expectation. The simulation test yielded an empirical *P*-value, calculated as the proportion if the observed count of cgSNPts in the 500 eQTLs exceeds the background level (the expected counts of cgSNPts in simulated SNP sets. The same simulation processes were conducted for trait-associated SNPs as well. The sampling times were dependent on the number of available proxy SNPs for sampling. Simulation tests were performed using SAS 9.2.

## SUPPLEMENTARY DATA, FIGURE AND TABLES


