# Supplementary material for: Polymorphisms involving gain or loss of CpG sites are significantly enriched in trait-associated SNPs
Source: Oncotarget. 2015 Oct 14;6(37):39995–40004. doi: 10.18632/oncotarget.5650 (PMC4741875; doi:10.18632/oncotarget.5650)
Supplement: Supplementary file 1 [file oncotarget-06-39995-s001.pdf]

SUPPLEMENTARY DATA , FIGURE AND TABLES

DETAILS OF TRAIT CATEGORIES

The traits/phenotypes were classified into different categories (including obesity and metabolic related, cancer related and neurological diseases) according to the 'Disease/Trait' label in the catalog of GWAS in NHGRI. We performed enrichment analyses in each categories separately after pooled analyses. The lists of traits/phenotypes in each categories are presented as follows:

OBESITY AND METABOLIC RELATED TRAITS

Adiponectin levels
Blood pressure
Body mass index
Body mass index (interaction)
Body mass index (non-asthmatics)
Coronary artery calcification
Coronary artery disease
Coronary artery disease or large artery stroke
Coronary heart disease
Fasting glucose-related traits
Fasting glucose-related traits (interaction with BMI)
Fasting insulin (interaction)
Fasting insulin-related traits
Fasting insulin-related traits (interaction with BMI)
Fasting plasma glucose
Fat body mass
Glycated hemoglobin levels
HDL cholesterol
Hypertension
Insulin-related traits
LDL cholesterol
Lipid metabolism phenotypes
Lipid traits
Metabolic syndrome
Obesity
Obesity (early onset extreme)
Proinsulin levels
Triglycerides
Two-hour glucose challenge
Type 1 diabetes
Type 2 diabetes
Waist circumference
Waist-hip ratio
Weight

NEUROLOGICAL DISEASES

Alzheimer's disease
Alzheimer's disease (late onset)
Anger
Autism
Bipolar disorder
Bipolar disorder (body mass index interaction)
Bipolar disorder (mood-incongruent)
Bipolar disorder and major depressive disorder (combined)
Bipolar disorder and schizophrenia
Depression (quantitative trait)
Eating disorders
Major depressive disorder
Major depressive disorder (broad)
Migraine with aura
Parkinson's disease
Psychosis (atypical)
Response to antipsychotic treatment
Response to antipsychotic treatment in schizophrenia (working memory)
Schizophrenia

CANCER RELATED

Acute lymphoblastic leukemia (B-cell precursor)
Acute lymphoblastic leukemia (childhood)
Bladder cancer
Bladder cancer (smoking interaction)
Breast cancer
Breast cancer (early onset)
Breast cancer (prognosis)
cancer(clinvar)
Chronic lymphocytic leukemia
Colorectal cancer
Colorectal cancer (diet interaction)
Endometrial cancer
Erectile dysfunction and prostate cancer treatment
Esophageal adenocarcinoma
Esophageal cancer
Esophageal cancer (alcohol interaction)
Esophageal cancer (squamous cell)
Esophageal cancer and gastric cancer
Esophageal squamous cell cancer (length of survival)
Essential tremor
Estradiol plasma levels (breast cancer)
Gallbladder cancer
Gastric cancer
Lobular breast cancer (menopausal hormone therapy interaction)
Lung cancer
Lung Cancer (DNA repair capacity)
Lung cancer (smoking interaction)
Lung cancer-asbestos exposure interaction
Melanoma
Multiple myeloma

Multiple myeloma (hyperdiploidy)
Multiple myeloma (IgH translocation)
Myeloproliferative neoplasms
Non-melanoma skin cancer
Non-small cell lung cancer
Non-small cell lung cancer (recurrence rate)
Non-small cell lung cancer (survival)
Osteosarcoma
Ovarian cancer
Pancreatic cancer
Prostate cancer
Prostate cancer (early onset)
Prostate cancer (gene x gene interaction)
Renal cell carcinoma
Response to antineoplastic agents
Response to irinotecan and platinum-based chemotherapy in non-small-cell lung cancer
Response to irinotecan in non-small-cell lung cancer
Response to platinum-based chemotherapy in non-small-cell lung cancer
Response to radiotherapy in cancer (late toxicity)
Response to tamoxifen in breast cancer
Small-cell lung cancer (survival)
Testicular cancer
Testicular germ cell cancer
Testicular germ cell tumor
Thyroid cancer
Tumor biomarkers
Upper aerodigestive tract cancers
Urinary bladder cancer
Urinary symptoms in response to radiotherapy in prostate cancer
Wilms tumor

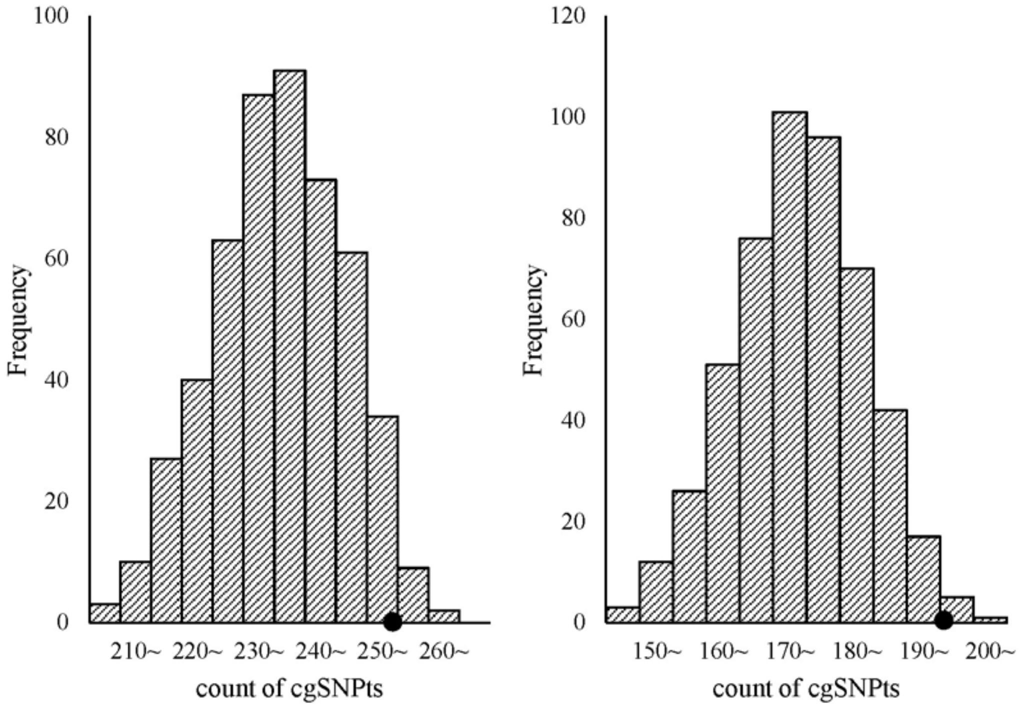

Supplementary Figure S1: cgSNPts are significantly enriched in trait-associated loci in European ancestry populations. The theoretical distribution of the count of cgSNPts in 500 draws (each draw containing 363 SNPs, which matched to the 363 trait-associated loci) are shown in the left bar graphs. The observed counts of cgSNPts in the 363 trait-associated loci was shown as a solid circle. 254 of the 363 variants, higher than the count from matched 500 times sampling (P=0.026) were cgSNPt in CEU populations. The right bar graph showed the distribution of the counts of cgSNPts in 500 draws. Each draw contained 285 SNPs, which matched to 285 cancer-associated loci. The count of cgSNPts in 285 cancer associated SNPs was significantly higher than the counts in matched draws (count=195, P=0.016).

Supplementary Table S1: The 42 scenarios of single base substitution that could cause gain or loss of a CpG site

Scenarios*	Count	Proportion(%)	Scenarios*	Count	Proportion(%)
CRT	160515	17.03	CDT	907	0.10
AYG	159549	16.93	AHG	906	0.10
CRG	85353	9.06	CVT	847	0.09
CYG	84702	8.99	ABG	830	0.09
CRA	71202	7.56	GHG	483	0.05
TYG	70563	7.49	CDC	475	0.05
GYG	64330	6.83	TBG	473	0.05
CRC	64258	6.82	CVC	463	0.05
CST	19023	2.02	CVA	457	0.05
ASG	18873	2.00	GBG	441	0.05
CKT	15188	1.61	CHG	438	0.05
AMG	15128	1.61	CDG	425	0.05
CKG	12702	1.35	CBT	345	0.04
CMG	12403	1.32	CDA	309	0.03
CSA	12227	1.30	AVG	308	0.03
TSG	11894	1.26	THG	291	0.03
CSC	10559	1.12	GVG	158	0.02
GSG	10420	1.11	CBC	144	0.02
CKC	9825	1.04	TVG	132	0.01
GMG	9734	1.03	CBA	113	0.01
CKA	7539	0.80			
TMG	7497	0.80			

*the middle base was coded by the International Union of Pure and Applied Chemistry (IUPAC) code.

Supplementary Table S2: The basic information of the 53 point mutations (cgMut), which involving loss of CpG sites, observed in tumor tissue only and the methylation levels of the 53 involved CpG sites in tumor and paired normal tissue sample (TCGA ID=3518)

Chromo-some	Position (hg19)	Gene	Reference Allele	Normal Allele1	Normal Allele2	Tumor Allele1	Tumor Allele2	Methylation level in normal tissue	Methylation level in tumor tissue
1	9780920	*PIK3CD*	C	C	C	C	T	81.8%	18.2%
1	10239537	*UBE4B*	G	G	G	G	A	100.0%	72.7%
1	19062164	*PAX7*	G	G	G	G	A	88.2%	41.2%
1	63021531	*DOCK7*	C	C	C	C	T	100.0%	66.7%
1	111660810	*DRAM2*	C	C	C	C	T	100.0%	64.3%
1	211545629	*TRAF5*	C	C	C	C	T	100.0%	82.4%
2	68607476	*PLEK*	C	C	C	C	T	100.0%	66.7%
2	103334923	*MFSD9*	G	G	G	G	A	100.0%	68.0%
2	170092504	*LRP2*	C	C	C	C	T	93.1%	84.3%
2	211476996	*CPS1*	G	G	G	G	A	100.0%	76.2%
3	31743923	*OSBPL10*	G	G	G	G	A	85.7%	85.7%
3	108363342	*DZIP3*	G	G	G	G	A	95.5%	91.3%
3	136287655	*STAG1*	C	C	C	C	T	92.3%	76.2%
3	172351414	*NCEH1*	G	G	G	G	A	81.5%	78.1%
3	187447463	*BCL6*	G	G	G	G	T	94.1%	87.5%
4	17826619	*NCAPG*	C	C	C	C	T	100.0%	61.9%
4	153249385	*FBXW7*	G	G	G	G	A	94.1%	80.0%
4	153809415	*ARFIP1*	C	C	C	C	T	96.6%	70.6%
5	40853769	*CARD6*	C	C	C	C	T	82.4%	73.7%
5	133914580	*PHF15*	G	G	G	G	A	100.0%	60.0%
6	11185508	*NEDD9*	C	C	C	C	T	93.8%	78.1%
6	31696455	*DDAH2*	G	G	G	G	A	84.6%	60.0%
6	39158947	*KCNK5*	C	C	C	C	T	95.7%	85.7%
6	43039980	*KLC4*	G	G	.	G	A	86.4%	76.2%
6	161807881	*PARK2*	G	G	G	G	A	100.0%	66.7%
7	2834685	*GNA12*	G	G	G	G	A	86.4%	58.1%
7	51096959	*COBL*	G	G	G	G	A	90.5%	77.8%
8	30969188	*WRN*	C	C	C	C	T	96.6%	93.1%
8	33451068	*DUSP26*	G	G	G	G	A	87.5%	57.1%
9	33465245	*NOL6*	C	C	C	C	T	100.0%	63.6%
10	33200929	*ITGB1*	G	G	G	G	A	100.0%	77.6%

(*Continued*)

Chromosome	Position (hg19)	Gene	Reference Allele	Normal Allele1	Normal Allele2	Tumor Allele1	Tumor Allele2	Methylation level in normal tissue	Methylation level in tumor tissue
11	104819264	*CASP4*	C	C	C	C	T	88.9%	83.9%
11	123676717	*OR6M1*	G	G	G	G	A	95.5%	80.0%
12	7522247	*CD163L1*	G	G	G	G	A	88.9%	65.2%
12	53552447	*CSAD*	G	G	G	G	A	92.9%	72.7%
12	69652698	*CPSF6*	G	G	G	G	A	100.0%	70.5%
12	120984350	*RNF10*	C	C	C	C	T	100.0%	96.0%
13	26789264	*RNF6*	C	C	C	C	T	100.0%	90.0%
13	114766383	*RASA3*	G	G	G	G	A	90.9%	85.7%
15	90176163	*KIF7*	G	G	G	G	A	83.3%	57.1%
16	50134200	*HEATR3*	C	C	C	C	T	100.0%	63.2%
16	70595628	*SF3B3*	G	G	G	G	A	95.2%	79.2%
16	71149659	*HYDIN*	C	C	C	C	T	66.7%	71.4%
17	2186924	*SMG6*	G	G	G	G	A	95.2%	68.2%
17	3977627	*ZZEF1*	C	C	C	C	T	77.1%	63.0%
17	10354747	*MYH4*	C	C	C	C	T	100.0%	50.0%
19	40433201	*FCGBP*	G	G	G	G	A	84.6%	59.1%
19	54818815	*LILRA5*	G	G	G	G	A	95.2%	75.0%
20	3575196	*ATRN*	C	C	C	C	T	91.2%	87.5%
20	4842674	*SLC23A2*	C	C	C	C	T	93.3%	71.4%
20	32298470	*PXMP4*	C	C	C	C	T	92.0%	65.0%
21	40600468	*BRWD1*	G	G	G	G	A	70.6%	85.0%
22	29745285	*AP1B1*	G	G	.	G	A	100.0%	80.0%
